# Action of Thyroid Hormones, T3 and T2, on Hepatic Fatty Acids: Differences in Metabolic Effects and Molecular Mechanisms

**DOI:** 10.3390/ijms18040744

**Published:** 2017-03-31

**Authors:** Fabrizio Damiano, Alessio Rochira, Antonio Gnoni, Luisa Siculella

**Affiliations:** 1 Laboratory of Biochemistry and Molecular Biology, Department of Biological and Environmental Sciences and Technologies, University of Salento, 73100 Lecce, Italy; alessio.rochira@unisalento.it (A.R.); luisa.siculella@unisalento.it (L.S.); 2 Department of Basic Medical Sciences, Section of Medical Biochemistry, University of Bari Aldo Moro, 70125 Bari, Italy; Antonio.gnoni@uniba.it

**Keywords:** 3,5-diiodo-l-thyronine, 3,5,3′-triiodo-l-thyronine, acetyl-CoA carboxylase, citrate carrier, de novo lipogenesis, fatty acid synthase, lipid lowering action

## Abstract

The thyroid hormones (THs) 3,3′,5,5′-tetraiodo-l-thyronine (T4) and 3,5,3′-triiodo-l-thyronine (T3) influence many metabolic pathways. The major physiological function of THs is to sustain basal energy expenditure, by acting primarily on carbohydrate and lipid catabolism. Beyond the mobilization and degradation of lipids, at the hepatic level THs stimulate the de novo fatty acid synthesis (de novo lipogenesis, DNL), through both the modulation of gene expression and the rapid activation of cell signalling pathways. 3,5-Diiodo-l-thyronine (T2), previously considered only a T3 catabolite, has been shown to mimic some of T3 effects on lipid catabolism. However, T2 action is more rapid than that of T3, and seems to be independent of protein synthesis. An inhibitory effect on DNL has been documented for T2. Here, we give an overview of the mechanisms of THs action on liver fatty acid metabolism, focusing on the different effects exerted by T2 and T3 on the regulation of the DNL. The inhibitory action on DNL exerted by T2 makes this compound a potential and attractive drug for the treatment of some metabolic diseases and cancer.

## 1. Introduction

Thyroid hormones (THs) are represented by 3,3′,5,5′-tetraiodo-l-thyronine (or thyroxine, T4) and 3,5,3′-triiodo-l-thyronine (T3). Thyroid gland produces mainly T4, whereas the bulk of the systemic T3, which is considered the most potent thyroid hormone, is generated by the deiodination of T4 in peripheral tissues. Three types of deiodinases exist: the type I (D1) is present in peripheral tissues, including liver; the type II (D2) is mainly expressed in the pituitary gland, brain, and brown adipose tissue; and the type III (D3) is present in placenta, brain, and skin [1]. It is generally accepted that D1 and D2 contribute to the conversion of most of T4 into T3.

In the blood, THs are mainly bound to specific proteins, which transport them through the circulation; only a small part of THs is free and exerts its action on target tissues. THs have profound effects on many physiological processes, such as development, growth and metabolism; defects in their production are not compatible with health. THs control the metabolism of proteins, carbohydrates and lipids. Increased levels of THs stimulate fat mobilization from adipose tissue, leading to raised concentrations of free fatty acids in the plasma. THs also enhance oxidation of fatty acids in many tissues, and affect cholesterol metabolism as well [2]. T3 and some THs mimetic compounds reduce the plasma concentration of cholesterol, by inducing its hepatic uptake and conversion into bile acids and by favouring faecal bile acid excretion [3,4]. In liver, gluconeogenesis and glycogenolysis are promoted by THs in order to generate free glucose [5,6], whereas in peripheral tissues THs stimulate the insulin-dependent uptake of glucose into cells and the subsequent glycolysis [5,7]. THs enhance the synthesis as well as the degradation of proteins. Supraphysiological doses of THs cause the depletion of skeletal muscle protein stores and increase the activities of protein catabolizing lysosomal enzymes [8].

THs control the energy metabolism and mitochondria are considered possible subcellular loci of thyroid hormone action [9]. In the mitochondrion, the major site of oxidative processes, THs stimulate the oxygen consumption as well as the ATP hydrolysis [7], thus participating to the regulation of the basal metabolic rate (BMR) and to the body heat production. Extensive changes occur in the mitochondrial compartment in response to the thyroid state of the animal [9,10].

T3 can be further deiodinated to 3,5-diiodo-l-thyronine (T2). Several studies indicated that, in addition to T3, T2 exhibits important biological effects in liver as well as in other tissues (for a review see [11]). A great body of evidence has reported that T2, previously considered only a T3 catabolite, is able to mimic some of T3’s effects on the metabolism [11]. T2 action appears to be more rapid than that of T3, and seems to be independent of protein synthesis [11,12,13,14,15]. It has been shown that T2 administration to rats increases their resting metabolic rate by modulating mitochondrial function [16,17,18], prevents diet-induced obesity by enhancing burning of fats [19], and reduces liver steatosis in rats fed on a high-fat diet [20]. T2 is also able to reduce the fat storage excess by acting directly on liver cells [21]. Lanni and colleagues [19] reported that action of T2 occurs without unfavourable side effects (i.e., thyrotoxicosis) usually observed when T3 or T4 is administered to rats [22].

Moreover, T2 is able to influence mitochondrial activities. It stimulates the mitochondrial oxidative capacity and respiration rate [9,11,14,15,23] and increases FoF1-ATP synthase expression and activity in rat liver mitochondria [14,24].

## 2. Genomic and Nongenomic Mechanisms of Action of Thyroid Hormones

THs play fundamental roles in the regulation of cell functions through genomic and nongenomic actions.

### 2.1. Genomic Actions

The classic, genomic mechanism of THs action requires the involvement of nuclear thyroid receptors (TRs), bound to the promoter of target genes. TRs are DNA-binding proteins that function as hormone-responsive transcription factors with a mechanism similar to that of receptors for steroid hormones [25]. Thyroid hormone receptor-α1 (TRα1) and thyroid hormone receptor-β1 (TRβ1)/thyroid hormone receptor-β2 (TRβ2) are encoded by *Thra* and *Thrb* genes, respectively. Each TR forms a heterodimer with RXR, or a homodimer with another thyroid receptor, which binds to DNA in a T3-independent way [25]. In vitro experiments suggest that the binding of TRs could occur on responsive elements constituted by doublets of the half-site AGGCTA, or by DNA elements bearing variations in sequence, spacing and orientation of this half site [26]. T3 and T4 enter cells through several membrane transporter proteins, some of them require ATP hydrolysis for the transport activity [27]. Once inside the nucleus, THs interact with the receptor bound to the promoter of the target genes, modulating their expression, either by stimulating or by inhibiting transcription [25].

### 2.2. Nongenomic Actions

Beyond the genomic effects, THs have been shown to initiate non-genomic signalling, which has been described at plasma membrane level, in cytoskeleton, in cytoplasm [28,29,30] and in various organelles, such as mitochondria [20]. These signals include alterations in transport of Ca^2+^, Na^+^ and glucose, as well as changes in activities of several kinases such as protein kinase C (PKC), phosphoinositide 3-kinase (PI3K), and mitogen-activated protein kinase (MAPK) [29,30].

The non-genomic actions of THs require a plasma membrane receptor or thyroid receptors located in cytoplasm [29]. The plasma membrane receptor is located on the integrin αvb3 at the Arg-Gly-Asp recognition site, important for the binding of extracellular matrix proteins [31,32,33]. Acting at the integrin receptor and without cell entry, T4 can foster ERK1/2-dependent serine phosphorylation of TRβ1, activating it [34]. In rat skeletal muscle T3 rapidly activates the protein kinase B (PKB)/Akt pathway, resulting in the stimulation of both fatty acid and glucose metabolism [35].

In human fibroblasts the PKB/Akt pathway is implicated in the transduction of the thyroid hormone signal, culminating in the activation of specific transcription factors [36]. It has been reported that the PKB/Akt transduction pathway requires the functional interaction between cytoplasmic TRβ1 and PI3K. This interaction causes the activation of PKB/Akt, which in turn induces the mTOR pathway implicated in the stimulation of protein synthesis [36]. Finally, T3 induces PKC activation in human hepatoma cells [29], in rat liver slices and in isolated hepatocytes [37].

## 3. Fatty Acid Metabolism in Liver

Metabolism of fatty acids (FA) is largely recognised as a target of THs action. Liver plays a crucial role in regulating fatty acid metabolism either in catabolic and anabolic direction. In liver free FA can have different sources:

(i) can be synthesized directly within the hepatocytes through the involvement of de novo lipogenesis (DNL),
(ii) can be taken up by liver from the pool of plasma FA released by the adipose tissue,
(iii) can be generated in liver from the hydrolysis of chylomicrons coming from intestine.

The metabolic fate of FA in liver is regulated by the nutritional/hormonal status of the organism: when energy request is low, hepatic FA are esterified into glycerol and stored as triacylglycerols (TAGs) or secreted in the plasma as very low density lipoprotein (VLDL). Vice versa, when energy request occurs, FA are oxidized mainly at mitochondrial level through the β-oxidation pathway. Liver mitochondria play a strategic role both in fatty acid β-oxidation, the major process by which FA can generate energy, as well as in DNL.

## 4. Mitochondrial Fatty Acid β-Oxidation

Fatty acid β-oxidation is localized in the mitochondrial matrix, while FA come from the cytosol. Short- and medium-chain FA freely enter mitochondria, while long-chain FA are preliminarily activated to acyl-coenzyme A by long chain acyl-CoA synthase, present in the outer mitochondrial membrane. Due to the impermeability of the inner mitochondrial membrane (IMM) to electrically charged molecules, the transfer of acyl-CoA across this membrane occurs through three consecutive steps: (1) the transfer of acyl group from acyl-CoA to carnitine, producing acylcarnitine, catalysed by carnitine palmitoyltransferase I (CPT I) at the external side of IMM; (2) the transport of acylcarnitine through the IMM by the carrier protein carnitine-acylcarnitine translocase; (3) the intramitochondrial reconversion of acylcarnitine to acyl-CoA ester by carnitine palmitoyltransferase II (CPT II). After that fatty acid β-oxidation may occur.

The main control of the rate of mitochondrial β-oxidation flux resides at the level of CPT I [38]. The major physiological inhibitor of CPT I is malonyl-CoA, the product of acetyl-CoA carboxylase, which catalyses the initial step of DNL.

Once in the mitochondrial matrix, the fatty acid β-oxidation process occurs through four individual reactions generating reducing equivalents in the form of FADH_2_ and NADH + H^+^, which are reoxidized to FAD and NAD^+^ by the mitochondrial respiratory chain, with the simultaneous production of ATP by the oxidative phosphorylation system.

The complete oxidation of acyl-CoA occurs through several cycles, shortening fatty acid of two carbons (i.e., acetyl-CoA) at each cycle. The end product acetyl-CoA is further metabolized in the tricarboxylic acid (TCA) cycle.

## 5. De Novo Lipogenesis (DNL) and Its Regulation

**Figure 1:**
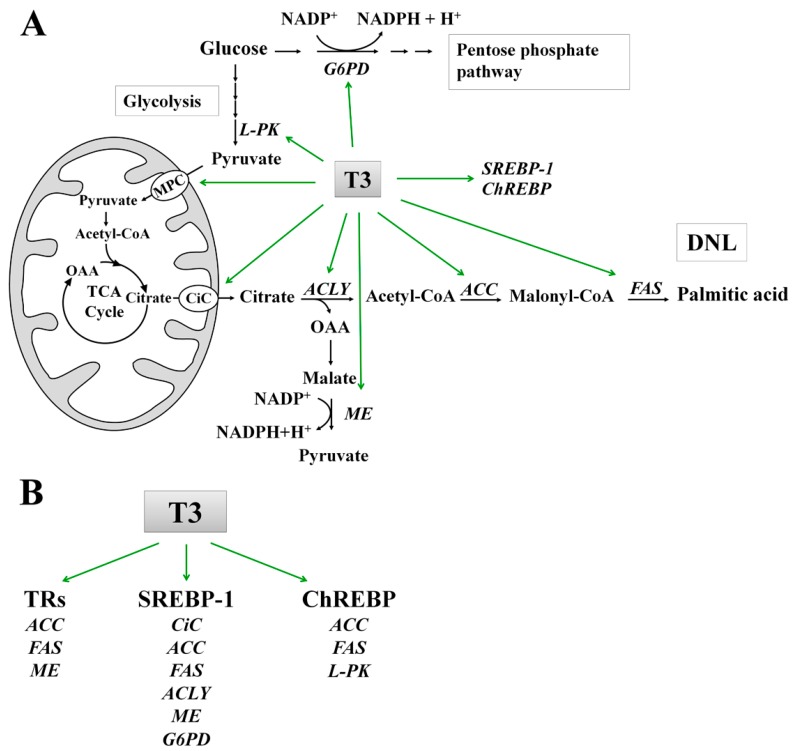
(**A**) Schematic representation of the role of T3 in the stimulation of activity and expression of enzymes, mitochondrial carriers, and transcription factors involved in the synthesis of palmitic acid from glucose; and (**B**) Effects of T3 on the activation of fatty acid synthesis through thyroid receptors (TRs) and lipogenic transcription factors (SREBP-1 and ChREBP). Abbreviations: ACC, Acetyl-CoA carboxylase; ACLY, ATP-citrate lyase; ChREBP, Carbohydrate Response Element Binding Protein; CiC, Citrate carrier; DNL, De novo lipogenesis; FAS, Fatty acid synthase; G6PD, Glucose-6-phosphate dehydrogenase; L-PK, Liver pyruvate kinase; MPC, Mitochondrial pyruvate carrier; ME, Malic enzyme; OAA; oxalacetic acid; SREBP-1, Sterol regulatory element-binding protein 1; TCA, Tricarboxylic acid cycle.

In this review we focused on THs effects on the DNL in liver. DNL is the metabolic pathway by which FA, primarily palmitic acid, are synthesized mainly from carbohydrates. The flow of carbons from glucose to fatty acids in the lipogenic pathway involves a coordinated series of enzymatic reactions. Glucose, taken up by the glucose transporter, enters the glycolytic pathway in the cytosol and generates pyruvate (Figure 1). Through mitochondrial pyruvate carrier (MPC), the pyruvate enters the mitochondrion [39], where, by the action of pyruvate dehydrogenase, it is converted into acetyl-CoA, which condenses with oxaloacetate to form citrate in the TCA cycle. When cellular energy is in excess, TCA cycle is inhibited, and the produced citrate exits the mitochondrion through the citrate carrier (CiC), localized in the IMM [40,41,42]. Once in the cytosol, by the action of ATP citrate lyase (ACLY), citrate is reconverted in oxalacetate (OAA) and acetyl-CoA, which represents the primer for DNL.

DNL is a cytosolic process catalysed by two multimeric enzymes working in sequence: (i) acetyl-CoA carboxylase (ACC) and (ii) the multienzymatic system of fatty acid synthase (FAS).

ACC catalyses the carboxylation, biotin dependent, of acetyl-CoA to form malonyl-CoA, which elongates the acyl chain by two carbons at a time, by a series of reactions catalysed by FAS. Palmitic acid (C16:0) is the main end product of this pathway. Palmitate can be further converted into longer and unsaturated fatty acids by fatty acid chain elongation and desaturation processes.

## 6. Regulation of Enzyme Activities of DNL

ACC is considered a key enzyme of DNL [43]. Its activity is inhibited by long chain acyl-CoAs, while it is positively modulated by citrate, which, as above reported, is exported from mitochondria by CiC. *ACC* gene transcription is under the control of multiple promoters, which are regulated by diet and hormones. The expression and the activity of ACC are reduced by starvation and glucagon whereas they are induced by insulin and fat-free diet rich in carbohydrates [44].

FAS is primarily expressed in the cytosol of liver, brain, adipose tissue, lung, and lactating mammary gland cells, where DNL is an important process. In mice or rats re-fed on a high-carbohydrate diet a robust induction of FAS expression, mediated by both insulin and glucose, was observed [45]. Conversely, glucagon and cyclic AMP inhibit the increase of FAS expression induced by carbohydrate re-feeding. Polyunsaturated fatty acids also exert a strong inhibition of FAS expression [45] and activity [46].

CiC plays an important role in the intermediary metabolism [41,42,46,47] and its activity is modulated by nutritional factors [46,48,49,50,51], and hormonal factors [52]. Moreover, the expression and the activity of CiC is affected by different pathological states [53,54,55,56].

Noteworthy, a covariance in the activities of CiC, ACC and FAS has been reported in different nutritional and hormonal states, thus highlighting a close correlation between mitochondrial (CiC) and cytosolic (ACC and FAS) reactions in FA biosynthesis [46,48,49]. In addition, high level of malonyl-CoA, the product of ACC, inhibits CPT I activity and decreases fatty acid β-oxidation; thereby, through malonyl-CoA, a coordination among hepatic FA synthesis, FA β-oxidation and ketogenesis occurs [43]. The hepatic neo-synthesized FA represent an important source for TAGs synthesis and hence for secretion rate of hepatic VLDL. Thus, hepatic DNL is closely correlated with plasma lipid concentration [57].

At transcriptional level, lipid synthesis is regulated by sterol regulatory element-binding proteins (SREBPs) [58] and carbohydrate response element binding protein (ChREBP) [59], which are considered the most important lipogenic transcription factors. SREBPs belong to a family of transcription factors including SREBP-1a, SREBP-1c and SREBP-2 that are involved in the control of lipid homeostasis through the regulation of several genes [58]. SREBPs are synthesized as inactive precursors (pSREBPs) bound to the endoplasmic reticulum (ER), where their regulatory domain interacts with SREBP-cleavage-activating protein (SCAP). SCAP functions as a sensor of membrane cholesterol levels. When an activation signal occurs, the SREBP-SCAP complex translocates from the ER to the Golgi apparatus, where a two-step proteolytic cleavage releases the N-terminal half of SREBPs (nSREBPs), allowing its entry into the nucleus. Conversely, in the absence of stimuli, the SREBP-SCAP complex remains in the ER membrane owing to its interaction with the ER-embedded insulin-induced gene (INSIG) protein [60].

ChREBP is a glucose-sensitive transcription factor controlling the conversion of carbohydrates into lipids in the liver [59]. ChREBP is a bZIP transcription factor that forms a heterodimeric complex with another bZIP protein max-like protein X (MLX) [59]. In low-glucose conditions, ChREBP is located in the cytosol and enters into the nucleus under high-glucose conditions [61].

## 7. Molecular Mechanisms of T3 Action on DNL

### 7.1. Effect of T3 on the Lipogenic Enzymes: ACC and FAS

DNL pathway is under the control of T3 [62] (Table 1). Within 4 h following the addition of T3 to monolayer cultures of hepatocytes isolated from eu- and hypothyroid rats, a very distinct stimulation of fatty acid synthesis and of FA incorporation into lipid fractions is observed [62]. However, stimulation of DNL by T3 is attenuated in rats fed high glucose diet supplemented with fats from beef tallow or safflower oil [63].

Different steps of DNL are increased in response to T3 administration [64]. T3 can modulate the expression of its target genes through two different ways: (i) directly, by the activation of thyroid receptor that interacts with the T3 responsive element (TRE) in the promoter region of the target genes [65,66,67]; (ii) indirectly, by activating transcription factors which in turn regulate the expression of target genes [68].

T3 is able to affect directly the two lipogenic enzymes ACC and FAS (Figure 1). T3 up-regulates ACC expression in chow-fed rats [69] through the TR bound to promoter of *ACC* gene [65,70]. In isolated chicken embryo hepatocytes, treatment with T3 increases the FAS mRNA [71]. In human hepatocarcinoma HepG2 cells, T3 activates FAS mRNA transcription through a TRE located in the FAS promoter [28]. A TR/RXR heterodimer binds the TRE even in the absence of the hormone, but the efficiency and the stability of this binding is increased in response to T3 [28]. T3 may also stimulate the expression of ACC and FAS via the transcription factor SREBP-1 [72]. Indeed, T3 up-regulates hepatic SREBP-1 [68] (Figure 1), which in turn stimulates the expression of *ACC* and *FAS* genes [73], through two adjacent SREBP-1 binding sites in their promoter. The expression of SREBP-1c is negatively regulated by T3 in chow-fed mice [74]. Experiments performed in vivo demonstrated that, when compared to control rats, SREBP-1 mRNA abundance was reduced in liver from T3-treated rats [30,75]. By contrast SREBP-1 protein amount was increased in hyperthyroid rats [30]. The molecular mechanism underlying the opposite effect of T3 on SREBP-1 mRNA versus the SREBP-1 protein in rat liver has not yet been investigated [30].

Recently, it has been evidenced in HepG2 that T3 is able to raise SREBP-1 protein level without affecting its mRNA level, enhancing the translation efficiency of its mRNA by an internal ribosome entry site (IRES)-mediated mechanism [30]. This effect is due to the non-genomic activation of both MAPK/ERK and PI3K/Akt pathways [30]. The administration of non-specific protein kinase inhibitors to cultured hepatocytes from embryonic chicken abolished T3-induced increase of the activity and expression of lipogenic enzymes FAS and ACC [76], showing a T3 action on signalling pathways.

*ACC* and *FAS* genes are positively regulated by ChREBP in response to glucose [59] (Figure 1). It is well demonstrated that in chow-fed mice T3 is able to activate ChREBP in liver (Figure 1) [77,78], suggesting the involvement of this transcription factor in the control of lipogenesis by THs.

### 7.2. Role of T3 in the Conversion of Carbohydrates into Fatty Acids

Two mitochondrial carrier proteins, modulated by T3, are involved in the conversion of carbohydrates into fatty acids: the mitochondrial carrier for pyruvate (MPC) and that for citrate (CiC) (Figure 1). The activity of MPC is enhanced in chow-fed hyperthyroid rats [79]. When compared to chow-fed euthyroid rats, CiC activity is severely down regulated in hypothyroid [53,54], and up regulated in hyperthyroid rats [80]. It has been demonstrated that hypothyroidism negatively affects the CiC expression at both transcriptional and post-transcriptional levels, through the inhibition of transcriptional rate of CiC mRNA and of its splicing [54].

T3 regulates the expression of some genes implicated in the glucose catabolism, in order to promote the production of acetyl-CoA and NADPH required for DNL (Figure 1). These genes include liver pyruvate kinase (*L-PK*) [81], *ACLY* [82], malic enzyme (*ME*) [83,84] and glucose 6-phosphate dehydrogenase (*G6PD*) [85].

In starved and high carbohydrate-refed rats, thyroid ablation did not alter the transcription of the gene for L-PK but caused a decrement in the L-PK mRNA content [81]. This suggests that T3 positively regulates L-PK expression at a post-transcriptional level [81]. However, L-PK is a well-known target of ChREBP, and a carbohydrate responsive element has been found in its promoter [86]. Thus, T3 may indirectly stimulate the L-PK expression through the activation of ChREBP [77]. In the vascular smooth muscle cell line, T3 controls the quaternary structure of the M2 isoform of PK (PKM2).

T3 reversibly binds to the monomeric and inactive form of PKM2, thus preventing the association of monomers to form the enzymatically active tetramer [87].

ACLY catalyses an important reaction linking glucose catabolism to DNL, by converting cytosolic citrate to oxaloacetate and acetyl-CoA, which is required for DNL and cholesterol synthesis. In cultured rat hepatocytes, T3 causes an increment of ACLY enzymatic activity [82], through stimulation of ACLY mRNA transcription [88] (Figure 1). The activation of ACLY expression by treatment with T3 is also observed during the differentiation of murine adipocytes [89]. Even though a TRE has not been found so far in the *ACLY* gene, T3 could induce the trans-activation of *ACLY* gene by SREBP-1, through a sterol regulatory element located in its promoter region [90].

ME catalyses the oxidative decarboxylation of malate to pyruvate with the concomitant production of NADPH, required for DNL (Figure 1). In hepatocytes isolated from chick embryo liver, T3 stimulates the ME activity as well as the transcription of the corresponding mRNA [76], by binding several TRE present in the 5′-flanking region of its gene [91]. When compared to chow-fed euthyroid rats, the level of ME mRNA was found to increase in hyperthyroid rats fed a high carbohydrate, fat-free diet [92]. The activation of ME expression by SREBP-1 has been also described [93]. T3 effects were reported for G6PD, another important NADPH producer that belongs to the pentose phosphate pathway (Figure 1). T3 is able to enhance hepatic G6PD activity in chow-fed rats [94,95], but remains unclear whether T3 directly induces *G6PD* gene expression [85,95]. It is well established, instead, the positive effect of SREBP-1 on the expression of G6PD [93].

There is another actor involved in the regulation of DNL by T3: Spot 14 (S14). *S14* is a family of genes which encode for two proteins S14 and S14-R, regulated by thyroid hormone and implicated in the control of lipid synthesis [96,97]. *S14* gene is highly regulated at the transcriptional level by SREBP-1c [98] and ChREBP [99].

Although DNL is increased in experimental models of hyperthyroidism, a reduced amount of plasma triacylglycerols and VLDL, as well as of hepatic intracellular TAGs was observed [100,101]. This can be explained, at least in part, by the increased FA oxidation observed in these models [100]. Beyond these mechanisms that counterbalance the increased hepatic TAGs synthesis, there is an extra-hepatic TH action that increases lipid clearance from the circulation, reducing serum lipid levels [90]. In hypothyroidism, however, concomitant with a reduction of FA synthesis [102] is also observed a FA oxidation decrease, which causes an increment of VLDL secretion by liver [103].

## 8. T2 and Its Effects on DNL

It has been reported that T2, previously considered only a T3 catabolite, is able to increase significantly the BMR, and greatly reduces adiposity and dyslipidemia without inducing unfavourable side effects (thyrotoxicosis) [17,19,104].

In rats fed on high fat diet, T2 prevents the pathways leading to lipid storage in lipid droplets (LDs), promotes the processes of lipid mobilization from LDs and secretion as VLDL [105] (Table 2). In in vitro model of hepatosteatosis, T2 stimulates the mitochondrial oxidation by up-regulating carnitine-palmitoyl-transferase (CPT1), uncoupling protein 2 (UCP2) and very long-chain acyl-coenzyme A dehydrogenase (VLCAD) [106]. By activating mitochondrial oxidative metabolism of fatty acids, in particular of saturated fatty acids, T2 reduces the number and size of LDs, and modifies their acyl composition by decreasing the content of saturated vs. monounsaturated fatty acids [106] (Table 2).

Unlike T3 whose action in stimulating lipogenesis is mainly mediated through TRβ, T2 has a reduced binding capacity to human TRβ [107] and shows a weak transactivating capacity of TRβ target genes in different systems [17,104,107].

**Figure 2:**
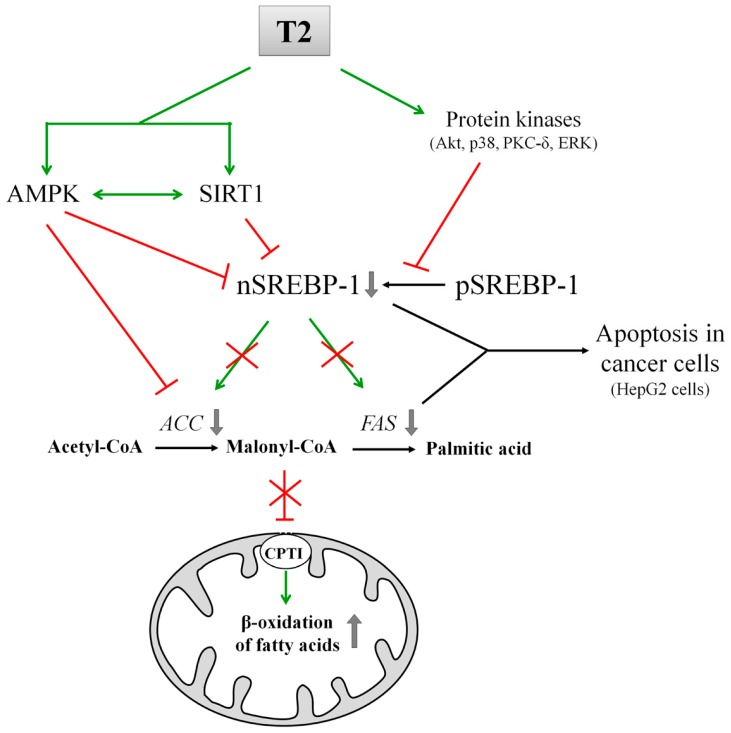
Scheme of the inhibition of fatty acid synthesis mediated by T2. Green line-arrow, Stimulation; Red T-bar, Inhibition; Red cross, Loss of stimulation/inhibition consequent to T2 action. Abbreviations: ACC, Acetyl-CoA carboxylase; AMPK, AMP-activated protein kinase; CPTI, Carnitine palmitoyltransferase I; FAS, Fatty acid synthase; nSREBP-1, nuclear sterol regulatory element-binding protein 1; pSREBP-1, precursor sterol regulatory element-binding protein 1; SIRT1, Sirtuin 1.

T2 carries out its antilipidemic effects by concomitantly activating fatty acid oxidation and down regulating lipogenesis [103,108] (Figure 2). This is achieved through the activation of two factors involved in lipid metabolism: AMPK and nuclear deacetylase sirtuin 1 (SIRT1) [103].

AMPK is a known detector of cellular ATP levels [109], and it acts as a master sensor of the energy status of the cell [110]. When activated via phosphorylation, AMPK phosphorylates, in turn, ACC that results inactivated: this state determines the down regulation of DNL (Figure 2); furthermore, the resulting reduced formation of malonyl-CoA causes the activation of CPT I and, as a consequence, the increase in fatty acid oxidation [111] (Figure 2).

Sirtuins are a group of histone/protein deacetylases regulated by variations in the cellular NAD^+^/NADH ratio. SIRT1, the most studied member of this family, responds to overfeeding, starvation, changes in energy expenditure and muscular exercise [112]. SIRT1 and AMPK can activate reciprocally, revealing the existence of an AMPK-SIRT1 cycle that links the cell energy to the redox states [112]. In addition, AMPK and SIRT1 (and most likely other sirtuins) act on common transcriptional activators and coactivators, including the mitochondrial master regulator PGC1α and members of the *FoxO* family [112]. It has been reported that T2 administration to high fat-fed rats determines a rapid and persistent activation of SIRT1 in liver [104,108] (Figure 2, Table 2). T2-activated SIRT1 catalyses the deacetylation of SREBP-1 and PGC1α causing their inactivation and activation, respectively. As a consequence of SREBP-1 inactivation, a down regulation of hepatic lipogenesis in high fat-fed rats was observed [104,108]. On the other hand, the SIRT1-mediated activation of PGC1α is correlated with the rapid induction of hepatic fatty acid oxidation in T2-treated rats [104,108]. Recently, the positive effects of T2 treatment on body lipid composition, characterized by the reduction of different fat depots and the increment of metabolic rate have been observed in obese mice [113]. However, the authors indicated that these positive effects were partially compromised by hyperphagia, which prevented weight loss in diet-induced obese mice [113]. It has been reported that T2, at pharmacological doses, inhibits lipogenesis in human HepG2 cells [114]. This effect is due to the block of the SREBP-1 activating cleavage, subsequent to the activation of ERK, p38, Akt and PKC-δ (Figure 2). The inhibition of SREBP-1 decreases, in turn, the expression of *FAS*, its target gene. Furthermore, all these cellular events, induced by T2, trigger caspase 3-dependent apoptosis of cultured HepG2 cells [115] (Figure 2).

Taken together, these findings suggest that T2 seems to be more effective with respect to T3 in lowering hepatic fat accumulation. Indeed, while T2 down regulates lipogenesis through SREBP-1 inhibition [104,114], T3 increases hepatic lipogenesis, even upon activation of SREBP-1 expression [29]. It has been shown that T2 prevents diet induced obesity [19], reduces liver steatosis in rodents [20], increases the resting metabolic rate and reduces body weight gain in humans [115]. Since T2 action occurs without thyromimetic side effects typically associated with T3 administration, T2 has been proposed as a potential hypolipidemic drug as well as a drug for the treatment of diet-induced obesity and hepatic steatosis in humans [115]. However, a very recent study raised concern about indiscriminate administration of T2 for the treatment of hyperlipidemia and pandemic obesity in humans [113]. Enlarged heart weights has been observed after T2 treatment, indicating potential cardiac side effects of this hormone [113]. Therefore, further studies are needed for a careful analysis of T2 mechanisms of action and safety.

## 9. Conclusions and Future Perspectives

The bulk of the literature about THs is focused on their effectiveness in reducing body weight, in counteracting visceral fat accumulation as well as hepatosteatosis, serum TAGs and cholesterol levels, and insulin resistance. Although THs can stimulate hepatic lipogenesis, prolonged THs treatment leads to the increase of FA oxidation and metabolic rate. For these reasons, it has been proposed the use of THs (T4 and T3) as therapeutic agents against obesity and correlated pathologies such as dyslipidemia, hepatic steatosis and cardiovascular disorders. The onset of thyrotoxic states (heart failure, loss of bone and muscle mass, fatigue) severely limits their clinical use, driving research in the development of TRs agonists and THs analogs with an adequate lipid-lowering and antiobesity efficacy, without thyrotoxic effects. In this context, growing is the interest for T2, able of an excellent lipid lowering action without the above mentioned side effects.

The goal of the present review was to focus the attention of the readers on T3 and its natural derivative T2, and on their action on liver fatty acid metabolism, highlighting the differences among them. T2 seems to be not properly a thyromimetic: while T3 stimulates both hepatic lipogenesis and fatty acid oxidation, T2 induces fatty acid oxidation and inhibits lipogenesis. Furthermore, as above described, there are other significant differences between the two hormones, concerning their molecular mechanisms of action.

Finally, it is interesting to correlate T2 ability to the down-regulation of lipogenesis and to the induction of apoptosis recently reported in HepG2 cells (Figure 2). The latter effect can be ascribed to the block of SREBP-1 proteolytic cleavage and to the FAS down regulation [114] (Figure 2). Accordingly, several in vivo and in vitro studies reported that pharmacological inhibition of SREBP-1 cleavage and of FAS activity, as well as silencing of FAS expression, induces apoptosis in cancer cells [116].

Indeed, FAS and SREBPs are considered key factors involved in neoplastic lipogenesis. Their overexpression is common to many cancers, and accumulating lines of evidence suggest that they are metabolic oncogenes with an important role in tumour growth and survival, making them attractive targets for cancer therapy [117]. Nowadays, there is a great interest for studies aimed at the development of new drugs and therapies to control the expression of FAS and SREBP-1. These observations highlight the possible use of the non-thyrotoxic hormone T2 as an inhibitor of lipogenic genes expression and activator of apoptosis, selectively in cancer cells.

## Figures and Tables

**Table 1:** Role and mechanism of action of T3 on activity or expression of enzymes and other proteins involved in fatty acid metabolism.

Experimental Model	Time of Treatment		Effect of T3 on Lipid Metabolism	Mechanism of Action	Ref.
In vitro studies					
HepG2 cells	24 h	↑	FAS mRNA	Genomic	[28]
HepG2 cells	Up to 24 h	↑	SREBP-1 protein synthesis	Non genomic	[30]
Hepatocytes from eu- and hypothyroid rats fed chow diet	4 h	↑	Synthesis of fatty acids and their incorporation into lipid fractions	Not reported	[62]
Cultured hepatocytes from chick embryo	Up to 49 h	↑	ACC promoter activity and mRNA abundance	Genomic	[66,67]
Cultured hepatocytes from chick embryo	Up to 48 h	↑	ME, FAS, ACC enzyme activity and mRNA abundance	Genomic	[76,71]
Hepatocytes from hypothyroid rats	24 h	↑	ACLY protein level and activity	Not reported	[82]
HepG2 cells	24 h	↑	ME promoter activity	Genomic	[83]
**In vivo studies**					
Liver from eu- and hyperthyroid rats fed chow diet.	4 weeks	↑	SREBP-1 protein level	Not reported	[30]
Liver from eu- and hypothyroid rats fed chow diet	4 weeks	↑	Mitochondrial citrate carrier expression, nuclear transcription rate and splicing efficiency	Genomic	[53,54]
Liver from eu- and hyperthyroid rats fed fat-enriched chow diet.	7 days	↑	Fatty acid synthesis	Not reported	[63]
Liver from hypo- and hyperthyroid rats fed chow diet.	7 days	↑	ACC mRNA abundance	Not reported	[69]
Liver from eu-, hypo- and hyperthyroid mice fed chow diet.	5 days	↓	SREBP-1 mRNA abundance and SREBP-1 promoter activity	Genomic	[74]
Liver from eu-, hypo- and hyperthyroid mice fed chow diet.	5 days	↑	ChREBP mRNA abundance and protein level; ChREBP promoter activity	Genomic	[77]
Liver from eu- and hyperthyroid mice fed chow diet or high carbohydrate diet.	Not reported	↑	ChREBP mRNA abundance; ChREBP promoter activity	Genomic	[78]
Liver from eu-, hypo-, and hyperthyroid rats, starved and refed on carbohydrate-rich diet	7 days	↑	G6PD enzyme activity	Non genomic	[85]
Liver from eu- and hyperthyroid rats fed chow diet or high carbohydrate, fat-free diet.	7 days	↑	ME mRNA abundance and enzyme activity	Not reported	[92]
Liver from eu- and hyperthyroid rats fed chow diet or high carbohydrate, fat-free diet.	7 days	↑	ME, G6PD and 6PGD enzyme activity, mRNA abundance and relative rate of enzyme synthesis	Genomic and non-genomic	[95]
Liver from hypo- and hyperthyroid rats fed chow diet	Up to 4 h	↑	Spot 14 protein (S14) mRNA abundance	Not reported	[96]
Liver from eu- and hyperthyroid rats fed chow diet	1 day	↑	Induction of citrate carrier activity	Not reported	[80]

↓ Decrease; ↑ Increase.

**Table 2:** Relevant effects of T2 on hepatic fatty acid metabolism.

Experimental Model	Time of Treatment	Effect of T2 on Lipid Metabolism	Ref.
In vitro studies			
FaO cells rendered steatotic by incubation of free fatty acids	24 h	Reduction in the number and size of lipid droplets in steatotic cells as consequence of triacylglycerols mobilization from lipid droplets. Stimulation of mitochondrial oxidative metabolism of fatty acids.	[106]
HepG2 cells	Up to 48 h	Induction of SREBP-1 proteolytic cleavage block and apoptosis in human hepatoma.	[114]
**In vivo studies**			
Liver from eu- and T2-treated rats fed chow diet or high fat diet	Up to 4 weeks	Reduction of hepatic fatty accumulation induced by a high-fat diet. Induction of fatty acid oxidation rate and of CPT I activity.	[20]
Liver from hypo-, eu- and T2-treated hypothyroid rats fed chow diet	1h	Increment of CPT-I activity and of total rate of fatty acid oxidation.	[15]
Liver from eu- and T2-treated rats fed chow diet or high fat diet	Up to 4 weeks	Deacetylation of peroxisome proliferator–activated receptor (PPAR)-γ and of SREBP-1 through the activation of SIRT1. Up-regulation of genes involved in the mitochondrial biogenesis and down-regulation of lipogenic genes.	[104]
Liver from eu- and T2-treated rats fed chow diet or high fat diet	30 days	Prevention of pathways leading to lipid storage in lipid droplets. Mobilization of lipids from lipid droplets and secretion as VLDL.	[105]

## Abbreviations

6PGD	6-Phosphogluconate dehydrogenase
ACC	Acetyl-CoA carboxylase
ACLY	ATP citrate lyase
AMPK	Protein kinase AMP-activated
BMR	Basal metabolic rate
ChREBP	Carbohydrate response element binding protein
CPT I	Carnitine palmitoyltransferase I
CPT II	Carnitine palmitoyltransferase II
CiC	Mitochondrial citrate carrier
DNL	De novo lipogenesis
ER	Endoplasmic reticulum
ERK1/2	Extracellular-signal regulated kinases 1/2
FA	Fatty acids
FAS	Fatty acid synthase
FoxO	Forkhead box O
G6PD	Glucose-6-phosphate dehydrogenase
IMM	Inner mitochondrial membrane
INSIG	Insulin-induced gene
IRES	Internal ribosome entry site
L-PK	Liver pyruvate kinase
LDs	Lipid droplets
MAPK	Mitogen-activated protein kinase
ME	Malic enzyme
MLX	Max-like protein X
MPC	Mitochondrial pyruvate carrier
mTOR	Mechanistic target of rapamycin
OAA	Oxalacetate
P38	P38 mitogen-activated protein kinase
PGC1α	Peroxisome proliferative activated receptor γ coactivator 1 α
PI3K	Phosphoinositide 3-kinase
PKB/Akt	Protein kinase B/serine/threonine kinase 1
PKC	Protein kinase C
PKM2	M2 isoform of pyruvate kinase
RXR	Retinoid X receptor
S14	Spot 14
SCAP	SREBP-cleavage-activating protein
SIRT1	Sirtuin 1
SREBPs	Sterol regulatory element-binding proteins
TAGs	Triacylglycerols
TRE	T3 responsive element
T2	3,5-Diiodo-l-thyronine
T3	3,5,3′-Triiodo-l-thyronine
T4	3,3′,5,5′-Tetraiodo-l-thyronine
TCA	Tricarboxylic acid
THs	Thyroid hormones
TRα1	Thyroid hormone receptor-α1
TRβ1	Thyroid hormone receptor-β1
TRβ2	Thyroid hormone receptor-β2
TRs	Thyroid receptors
VLDL	Very low density lipoprotein
